# Limited Potential of Repetitive Transcranial Magnetic Stimulation for Treatment of Essential Tremor: A Systematic Review

**DOI:** 10.3390/neurosci5040038

**Published:** 2024-11-05

**Authors:** Andrew A. E. D. Bishay, Anton Guo, Rhea Desai, Samuel Mushinski, Andy Au, Andrew J. Swenson, Marco Iacoboni, Alexander Bystritsky, Norman M. Spivak

**Affiliations:** 1 Physiological Sciences Interdepartmental Program, University of California, Los Angeles (UCLA), Los Angeles, CA 90095, USA; 2 Department of Psychiatry and Biobehavioral Sciences, University of California, Los Angeles (UCLA), Los Angeles, CA 90095, USA; 3 Neuroscience Undergraduate Interdepartmental Program, University of California, Los Angeles (UCLA), Los Angeles, CA 90095, USA; 4 Weill Cornell Medical College, Cornell University, New York, NY 10065, USA; 5 Department of Neurosurgery, University of California, Los Angeles (UCLA), Los Angeles, CA 90095, USA; 6 UCLA-Caltech Medical Scientist Training Program, University of California, Los Angeles (UCLA), Los Angeles, CA 90095, USA

**Keywords:** essential tremor, rTMS, transcranial magnetic stimulation

## Abstract

Essential tremor (ET) is a prevalent movement disorder characterized by action tremors, predominantly affecting the upper limbs. While various pharmacological and non-pharmacological interventions have shown efficacy in managing ET, the therapeutic role of repetitive transcranial magnetic stimulation (rTMS) remains uncertain. This systematic review synthesizes evidence from clinical trials investigating rTMS as a treatment for ET. Despite some open-label trials reporting reductions in tremor severity, double-blinded studies revealed no significant difference between active and sham rTMS, suggesting a strong placebo effect. The findings indicate that while rTMS can reduce tremor scores, its therapeutic efficacy in ET remains unproven. Future research should focus on improving sham designs and conducting larger, rigorously controlled trials to clarify rTMS’s role in ET management. Current evidence supports considering alternative treatments, such as deep brain stimulation, over rTMS for ET.

## 1. Introduction

Essential tremor (ET) is one of the most common movement disorders in the world, affecting up to 1% of the population and 5% of adults over 60 [1]. ET is a form of action tremor that impacts the upper limbs in at least 95% of patients and is associated with increased activity in the cerebello-thalamo-cortical circuit [2]. Typically, dysfunction of GABAergic signaling in the cerebellar dentate nucleus and brainstem—suggestive of neurodegeneration—drives tremor activity [3]. However, multiple explanations such as the neurodegeneration hypothesis (cerebellar degradation), GABA hypothesis (lowered GABAergic tone), and oscillating network hypothesis (hyperactive tremor network oscillators) may also explain ET’s heterogeneous pathophysiology [3]. A multifactorial combination of these pathogenic mechanisms likely plays a key role during the progression of ET, requiring a variety of treatment approaches for inducing neuroprotection, increasing GABAergic tone, and reducing rhythmic motor tremors in patients. Generally, symptoms are insidious and deteriorating, affecting patients’ posture, resting conditions, and daily actions.

### 1.1. Managing Essential Tremor

A wide body of evidence exists for ET treatments and measurements to evaluate treatment efficacy. Non-medical treatments such as adequate sleep and occupational therapy have shown some efficacy in remedying tremor symptoms [4,5]. Medical treatments such as propranolol, a non-selective β-adrenergic antagonist, can reduce limb tremor severity when used in young patients in the long-term, with a reported 50% of patients receiving benefit [6,7]. Primidone, a barbiturate anticonvulsant, appears to be more tolerable for the elderly, although its mean effect on tremor reduction is insufficient [8]. It can also cause drowsiness, which is a concern for the elderly, who are already at risk for falls. Recently, topiramate, an antiepileptic, has been recommended as a first-line treatment with robust efficacy in clinical trials but is known to produce cognitive side effects [9]. Regardless of drug class, adhering to published guidelines for drug usage is critical [7]. Alternatively, invasive treatments such as high-intensity focused ultrasound ablation have targeted the nucleus ventralis intermedius (Vim) of the thalamus and zona incerta, which have led to subsequent improvement in ET symptoms [10]. Focused ultrasound has strong evidence to support its reliable treatment quality, especially for unilateral ablation, with one randomized trial showing a reported 41% improvement in tremor score in the active group compared to 2% in the sham group [11]. Deep brain stimulation (DBS) also relies on similar targets to activate the cortico-thalamic projections at high frequencies [12], as stimulating the Vim and surrounding subthalamic structures has been shown to reduce tremor scores by 90% [7]. Radiosurgery has been performed in rare cases where focused ultrasound and DBS are not applicable despite their relative obsoleteness [13].

The success of ET treatments is often measured quantitatively through the Fahn–Tolosa–Marin Tremor Rating Scale (FTMTRS) and the Essential Tremor Rating Assessment Scale (TETRAS) [14]. FTMTRS records severe tremors based on maximal tremor amplitude, while TETRAS separates these values into corresponding body extremities. Other scales assess the quality of life from patient syndromes as a primary outcome, such as the Quality of Life in Essential Tremor Questionnaire (QUEST) [15]. Motion transducers such as accelerometers, albeit notably inconsistent with rating scales, have also been used as a treatment measure [7]. Nevertheless, motion transducers are still worthwhile as they provide useful data for treatment effects by measuring tremor amplitude [7].

Treating ET requires an all-around awareness of the patient’s unique symptoms and evaluation of specific treatment options that safely and effectively alleviate an individual patient’s tremor. Selecting an intervention also requires distinguishing among differential diagnoses, one of which is dystonic tremor, which shares overlapping conditions with ET. Educating patients about ET’s chronic characteristics and a multitude of non-medical options is a crucial part of an open-minded holistic approach. For instance, wearables are a promising implementation that may help deliver patient-centered care for ET patients by providing quality longitudinal clinical data [16]. Strikingly, one-third of patients who seek treatment stop their medication regimen, primarily due to mild side effects and a perceived lack of efficacy in the medication [17]. Lastly, seeking comprehensive evaluations from a movement disorders neurologist and functional neurosurgeon is imperative to understand all possible treatment options.

### 1.2. Clinical Perspective Consensus

Given that ET is an age-related progressive disease, brain modulation through non-invasive brain stimulation (NIBS) may improve the patient’s clinical outcomes [18].

Repetitive transcranial magnetic stimulation (rTMS) is a non-invasive brain stimulation (NIBS) technique that uses wire coils to generate magnetic fields via Ampere’s Law, which in turn produce electric currents in the brain tissue via Faraday’s Law, leading to changes in cortical excitability. The exact mechanism that underlies rTMS’s therapeutic effects is not entirely clear, but it is believed to act by mainly modulating the inferior olive and cerebellum of the oscillatory network or affecting synaptic plasticity [19,20]. Importantly, the intervention duration outlasts the period of stimulation [21]. Stimulation frequency, pulse number, and stimulation period determine the nature of the stimulation and its after-effects. rTMS has garnered support for its beneficial effects in treating patients with drug-resistant major depressive disorder, obsessive–compulsive disorder, and smoking addiction [20]. Generally, in the context of M1 stimulation, stimulation < 1 Hz (LF-rTMS) transiently reduces cortical excitability as motor-evoked potential (MEP) size decreases, and stimulation > 5 Hz (HF-rTMS) increases cortical excitability as MEP size increases [22,23,24]. With a focal stimulation area of 25 mm^2^, rTMS targets neural circuitry with some precision [25]. Other patterned protocols, such as theta burst stimulation (TBS) and quadripulse stimulation, also modulate brain activity [18]. For example, applying intermittent TMS (iTBS) with 600 pulses of TBS as 2-s trains cycled per 10 s mimics LTP-like plasticity in the M1, while applying continuous TBS trains for 40 s (cTBS) mimics LTD-like decrease in M1 excitability [26].

rTMS application on the motor cortex (M1) or posterior cerebellum has frequently been demonstrated to reduce ET symptoms [27]. M1 is often targeted because it allows cortical changes to be easily measured. HF-rTMS of M1 has a definitive analgesic effect when applied contralaterally for neuropathic pain, which has shown success in treating Parkinson’s symptoms [24,28]. Cerebellar stimulation is also common since rTMS may alleviate tremulous behavior associated with a dysfunctional cerebello-thalamo-cortical network [29,30]. 

### 1.3. Rationale for Review

While the demand for large-scale, double-blinded controlled trials remains, available evidence supports rTMS’s efficacy in alleviating ET symptoms. Recent studies show that at least 20–30 sessions are needed for optimal effects [31,32]. Typically, longer stimulation results in a longer duration of positive after-effects [31]. A previous open-label trial showed that five sessions a week of 1 Hz cerebellar rTMS for 3 weeks yielded modest improvement in tremor ratings [33]. A real vs. sham study saw significant tremor score reductions (26% and 19%, respectively) with clinical benefit at the 4- and 8-week follow-ups in the real rTMS arm [34].

Thus far, published rTMS studies on ET have been conducted on heterogeneous patient populations. The individualized patient-specific rTMS protocols to optimize treatment make it difficult to compare across patients, stimulation sites, and stimulation protocols [35]. Given the complex neurochemical changes underlying ET pathophysiology, clinical heterogeneity, and diagnostic uncertainty, more potent NIBS therapies are required to supplement this gap in treatment quality [36]. The evidence regarding ET’s efficacy is variable and fragmented, necessitating a comprehensive assessment of existing research. For these reasons, the present review synthesized evidence from existing clinical trials to assess ET symptoms’ response to rTMS and evaluate its efficacy as a treatment.

## 2. Materials and Methods

### 2.1. Protocol and Registration

This systematic review aimed to synthesize existing research to provide a comprehensive assessment of rTMS’s effectiveness and safety for ET, guide clinical practice, and identify areas for future research. The main goal of this systematic review was to analyze how effective rTMS is as a treatment for ET, including which parameters and targeting have been most efficacious in trials using rTMS to treat ET, how many clinical trials have used rTMS as a treatment for ET, and how the symptoms of ET respond to rTMS. The PubMed database was searched for relevant studies from the inception of the database to 27 February 2024. Articles considered were in English. The search strategy used involved the keywords “Essential Tremor”, “Repetitive Transcranial Magnetic Stimulation”, or “rTMS”. This systematic review protocol was registered with PROSPERO before starting the review PROSPERO CRD42024516823.

### 2.2. Eligibility Criteria

**Figure 1:**
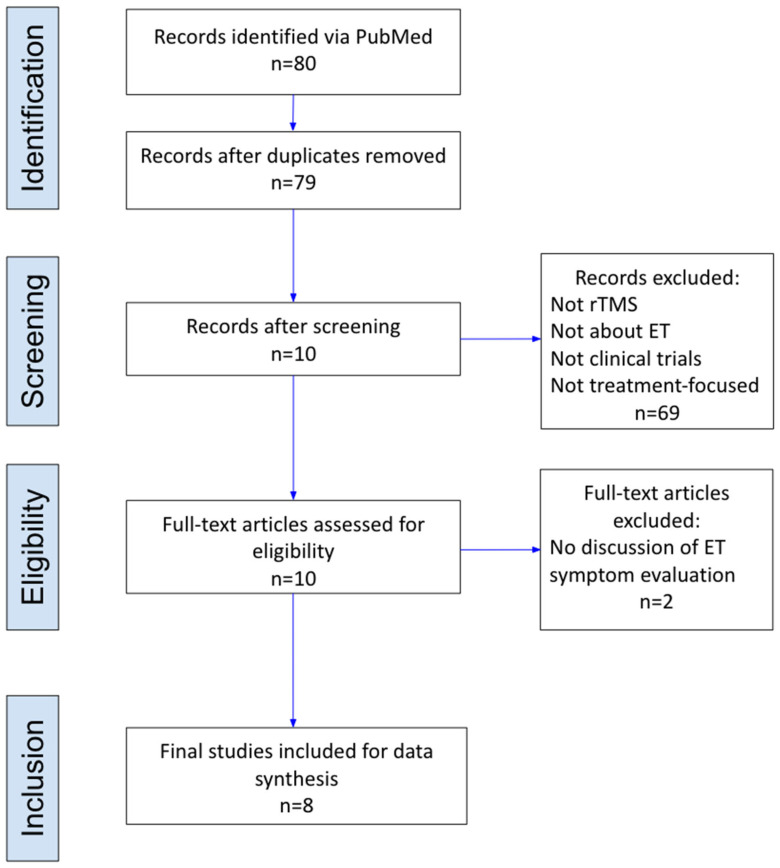
PRISMA flow chart of the publication selection process.

Articles considered were clinical trials in which rTMS was specifically employed as a treatment for ET. This ensured that the analysis was centered on the efficacy and outcomes of rTMS as a therapeutic intervention for ET. The initial PubMed database search resulted in 79 papers, of which 10 were selected and 2 were mediated for exclusion, for a total of 8 papers meeting the criteria. Randomized control trials (RCTs), single-blinded, double-blinded, and open-label studies in which ET patients underwent rTMS treatment were included. Excluded papers were those that did not use rTMS, were not relevant to ET, were not clinical trials, and did not focus on treating ET. This included any reviews, systematic reviews, or letters (Figure 1).

### 2.3. Study Selection and Data Collection Process

Four reviewers (A.A.E.D.B., A.G., R.D., and S.M.) applied these eligibility criteria and selected studies for inclusion, working in teams of 2 independently and blindly of the other. Both teams then presented the studies they determined to be eligible, with any differences in included studies reviewed and mediated by a fifth member (AA) who was not involved in either team’s selection process. Both teams and the mediator recorded their eligibility progress separately, which was then shared with the sixth member of the review team (AS), who was neither involved in the selection nor the mediation process. Study design, sample size, subject demographics, TMS targeting parameters, initial and final ET severity, and adverse events were extracted from each included study and recorded by the review team.

### 2.4. Patient Demographics

The general patient population was adults with ET whose symptoms had not responded to medications or other therapeutics. All studies employed the Movement Disorder Society (MDS) criteria for ET diagnosis. One study that used the old MDS criteria found that all but 1 subject would have fit the new criteria upon retrospective analysis [37].

Patients with other forms of tremor disorders or neurological signs of uncertain significance such as memory issues, impaired gait, and other signs were generally excluded during recruitment across trials. Patients with a seizure history or metallic implants such as cardiac pacemakers were also generally excluded. 

Across all included studies, 101 patients enrolled in trials received rTMS treatment. Some studies had larger sample sizes that were not reflected in this total, as some subjects served as healthy controls in open-label trials [38,39,40] or were randomly assigned to receive other non-rTMS treatments [41]. 

## 3. Results

### 3.1. Safety

Most trials reviewed had no adverse events reported in the rTMS groups. Only two of the studies reported any adverse events, mostly mild cases. However, one study had two patients drop out after receiving rTMS due to a lack of perceived efficacy [34]. One additional study reported patient dropout, with one patient leaving this study due to dizziness after the first active rTMS session [37]. A total of 14 adverse events were reported across the 101 patients who received rTMS, 12 of these coming from one study that had a sample size of 23 patients [37]. These events included reports of dizziness, headaches, neck spasms, and other mild incurrences. It is worth noting that of the four total cases of headaches reported, only two were after active rTMS, with the other two reported during sham rTMS administration [37]. However, more severe adverse events were also reported, including a transient ischemic attack (TIA)-like event and photopsia. One instance of a (TIA) like-event was reported during sham rTMS, resulting in 2 h of grade 4 left hemiparesis [37]. The hemiparesis was resolved independently, but no further or long-term follow-up was reported. One case of photopsia was specifically reported alongside two other cases of general visual disturbances, all reported during active rTMS [37,42]. Overall, rTMS is a safe treatment option for ET, with most adverse events being relatively mild and temporary. 

### 3.2. Targeting

The most common target for rTMS was the cerebellum, with five out of eight studies using cerebellar targeting procedures [33,37,39,41,42]. This resulted in 76 of the total 101 patients who underwent rTMS receiving cerebellar stimulation. The prominent rationale for cerebellum stimulation was the cerebellum’s role in ET’s pathogenesis [33,37]. Of these five studies, all five found significant improvement in ET symptoms as measured by a reduction in FTMTRS. However, four of these studies included sham controls, in which no sustained significance was found between sham and active rTMS [37,39,41,42]. One of these four studies found significance between active and sham rTMS 5 min after stimulation but did not find significance when measuring 60 min after treatment [42]. 

The other studies used motor area targets, with two studies targeting M1 [38,40], and one study instead targeting the pre-SMA [34]. Of these three studies, one of the studies targeting M1 and the study targeting the pre-SMA resulted in a decrease in FTMTRS scores [34,38], while the other M1 study only showed a decrease in ET symptoms by accelerometric measurements but not in clinical FTMTRS [40]. Of the two studies that resulted in FTMTRS reduction, one was a double-blinded study with sham rTMS control and found no between-group significance [34].

### 3.3. rTMS Parameters

Two different forms of rTMS studies were considered: traditional rTMS and cTBS, a patterned rTMS procedure [43]. Of the six studies utilizing traditional rTMS, all six used a pulse frequency of 1 Hz, but at differing intensities determined by the patient’s resting motor threshold (rMT). Four of these studies administered pulses at 90% rMT [33,37,39,41], one study at 100% rMT [42], and one study at 110% rMT [34]. Of the two cTBS studies, one used an intensity of 80% rMT [38], while the other study used an intensity of 80% of the active motor threshold (AMT) [40].

The length of stimulation and number of pulses also differed by study. Daily pulsation ranged from 400 to 1200 pulses per day. Generally, the rTMS protocol was administered daily for 5 days in a row [33,37,38,39], but one study continued stimulation for a total of 15 days, Monday through Friday, with weekends off for 3 weeks [34].

Five studies included sham or control rTMS [34,37,39,40,42]. Sham rTMS was designed such that stimulation could not produce a neuromodulatory effect, either by using placebo inactive coil systems [34,37] or by angling the coil 90° to the scalp such that it could not incite significant cortical excitation [39,42]. However, one study used cTBS at 30% AMT for its control as opposed to 80% AMT intensity in the active cTBS group, citing 30% AMT as an insufficient low-dose stimulation intensity [40].

### 3.4. Effectiveness

Seven out of eight studies showed an improvement in ET severity as classified by FTMTRS after active rTMS [33,34,37,38,39,41,42]. One study found no significant reduction in clinical tremor score for both active and control rTMS [40], but did find that active rTMS induced a significant reduction in tremor severity as measured by accelerometric ratings for at least 45 min post-stimulation (Table 1).

Table 1: This table summarizes the findings from various studies investigating the impact of rTMS on tremor severity as measured by the Fahn–Tolosa–Marin Tremor Rating Scale (FTMTRS) and the TETRAS. The studies vary in design, stimulation protocol, target region, sample size (n), and outcome measures.

There was limited between-group significance in trials with sham rTMS. In the three double-blinded studies [34,37,42], there was no overall between-group significance based on clinical ratings. However, one of these studies found clinical significance between sham and active rTMS tremor ratings only at 5 min post-stimulation [42]. This study also found a significant decrease in accelerometric values for active rTMS compared to sham, but also only at 5 min post-stimulation [41]. 

This trend was likewise observed in the one single-blinded study reported, in which the reduction in FTMTRS was not significant between active and sham rTMS [39].

Furthermore, one study examined the effects of rTMS versus propranolol in a single-blinded trial, producing a similar result in that rTMS resulted in a reduction in FTMTRS but was not significantly compared to propranolol treatment [41]. 

## 4. Discussion

**Figure 2:**
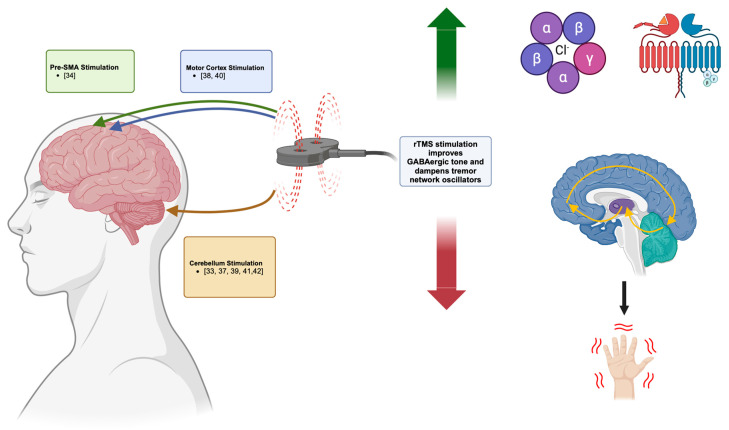
Pathophysiological application of rTMS on ET [33,34,37,38,39,40,41,42].

This systematic review examined the current literature to evaluate the therapeutic effectiveness of rTMS for ET. This review collected data and reported on the results from eight studies (Figure 2). Qualitative analysis of these studies was possible through the comparison of study results and qualitative conclusions on ET responsiveness to rTMS. However, while these studies all included quantitative metrics, the possibility of quantitative comparisons was limited due to a lack of published effect sizes. Generally, all studies used FTMTRS to clinically assess ET symptom changes in patients before and after stimulation. Also, some studies gave limited information on the actual FTMTRS subscores assessed, further limiting the possibility of conducting a meta-analysis beyond the systematic review.

Figure 2: This figure shows the different stimulation targets in the evaluated studies, showing the role of these targets as nodes in the cerebello-thalamo-cortical circuit. Current evidence points towards rTMS’s efficacy in alleviating ET symptoms by increasing GABAergic tone (GABA-A and GABA-B receptors shown in top right) and modulating the oscillatory network (cerebello-thalamo-cortical circuit shown in bottom right) to reduce tremors in the upper limbs. Created in BioRender. Guo, A. (2024) BioRender.com/h30e185.

Accelerometric ratings were used as a secondary metric to assess symptom improvement in some studies but not in others. Given that studies in which accelerometric metrics were taken did not provide specific outcome data and that accelerometric assessments are subject to significant intrasubject variability [42], these findings were not accounted for in the conclusions made in this systematic review.

The most significant finding across these eight studies was the lack of significance in higher-stringency trial designs. While rTMS was demonstrated to be clinically effective in reducing FTMTRS in open-label trials as well as producing a general reduction in FTMTRS from baseline in sham-controlled trials, there was a consistent lack of between-group significance for sham-controlled trials. This was true across the majority of single- and double-blinded studies. One study had significance at 5 min post-stimulation that was not maintained thereafter. The failure of this significance to persist in measurements taken after 5 min limits the applicability of this finding to validate rTMS as an effective therapeutic for ET, given that in clinical practice, tremor improvement would need to show sustained effectiveness. The other controlled study findings, in which FTMTRS improvements were reciprocated in both active and sham rTMS, suggest a strong placebo effect and lack of true therapeutic efficacy of rTMS for ET. 

A common concern of studies including sham rTMS is poor sham designs that fail to adequately replicate rTMS, thereby possibly conflating study outcomes and results [44]. One specific sham technique that has drawn criticism is the angling of the coil off of the scalp to direct the magnetic field away from the brain. It has been found that tilting the coil 45° can elicit cortical excitation anywhere from 48 to 76% of active rTMS excitation [45]. Tilting of the coil 90° has also been criticized, as the subjects are aware the coil is in a different position and sounds different [44]. Two of the studies reported sham designs in which the coil was tilted 90°, and none used the 45° method. While notably critiqued, if this design was to be a concern, we would have expected the sham groups to reflect a decreased reduction in FTMTRS given the possibility of a lack of perceived efficacy during sham stimulation, perhaps leading to pseudo-significance between sham and active groups. Given that these two studies still did not find a significant difference in tremor reduction between active and sham groups, sham design was likely not a point of concern in these studies. 

An improved sham design uses an inactive coil alongside simulated rTMS sounds and low-pulse electrical stimulation to induce scalp muscle twitching associated with active rTMS, which was utilized by an additional two studies in this review. Similarly, these studies also found no significance between FTMTRS improvement after active and sham rTMS, again suggesting the existence of a heavy placebo effect surrounding rTMS therapeutic effectiveness for ET. 

Alternatively, decreased intensity rTMS has been used as a form of control rTMS. It has been noted that these lower intensities may still induce substantial cortical stimulation [44]. Therefore, if the sham design is to elicit a strong effect, it can further validate the impact of active rTMS, as it may have been that only a minimal intensity of rTMS was required to produce the desired effects. Paradoxically, the only study reported with a control of this nature was the only study in which any significance between sham and active rTMS was found at 5 min post-stimulation. If the concern of only minimal rTMS neurostimulation being necessary to incite tremor reduction was applicable, we would have expected to see an inflated FTMTRS reduction in the sham leg, thus further limiting the significance between active and sham rTMS. Therefore, we conclude concerns over sham rTMS interference with FTMTRS reduction did not apply to this study nor this review, again validating the concerns of a placebo effect deduced from the other studies. 

## 5. Conclusions

In a systematic review of studies in which rTMS was tested as a therapeutic for ET, we have found that while being effective in reducing FTMTRS, rTMS failed to uphold the same efficacy in double-blinded trials. Specifically, the lack of comparative significance between active and sham rTMS success in tremor reduction suggests a lack of efficacy of rTMS as a therapeutic for ET. Furthermore, since different methodologies were used to arrive at chosen stimulation parameters across trials, and given the differences in parameters themselves, this further limits the generalizability of rTMS as an NIBS therapeutic approach for ET.

While alternative therapeutics should continue to be studied for ET, including those within the field of neuromodulation and neurostimulation such as DBS, other options, including traditional forms of treatment, should be considered more efficacious for ET than rTMS at this time. Further studies with larger sample sizes and infallible sham rTMS designs are needed to comprehensively conclude the inefficaciousness of rTMS for ET, but such trials are naturally unlikely given the lack of demonstrated efficacy in double-blinded studies thus far and the logistical challenges and expenses such designs would pose. 

## Figures and Tables

**Table 1:** Overview of studies evaluating the effectiveness of repetitive transcranial magnetic stimulation on tremor reduction in essential tremor (ET) patients.

Author and Year	Study Design	Stimulation Protocols (Active Groups)	Target Region	*n*	Outcome Measure	F and *p*<
[34]	Double-blinded, sham-controlled pilot trial	1200 daily pulses of 1 Hz rTMS at 110% rMT for 15 days	pre-SMA	5	FTMTRS	After 15 daily rTMS sessions, the active group showed a significant reduction in FTMTRS score (26.11% reduction, mean TRS decrease 9.4, SD 7.36, *p* = 0.0038). The sham condition also showed a reduction in FTMTRS score (18.82% reduction, mean TRS decrease 6.4, SD 4.615, *p* = 0.0497). Upon 4- and 8-week follow-ups, only the active group maintained significant decreases compared to baseline (17.77% decrease, mean point decrease 3, SD 2.64, *p* = 0.0497). Cohen’s *d* = 0.49.
[38]	Open-label trial	50 Hz cTBS triplets delivered every 200 ms for 40 s at 80% rMT	M1	10	FTMTRS and TETRAS	Significant reduction in tremor scores. FTMRS (Pre-cTBS: 29.3 ± 18.7, Post-cTBS: 25.3 ± 16.8; *p* < 0.001) and TETRAS (pre-cTBS: 34.4 ± 16.2, post-cTBS: 29.8 ± 12.1; *p* = 0.01).
[42]	Double-blinded, crossover, placebo-controlled	1 Hz at 100% rMT for 20 min daily for 5 days	Cerebellum	10	FTMTRS	FTMTRS scores were significantly lower at +5 min after active rTMS (20.7 ± 11.8) than after sham rTMS (23.4 ± 13.9) (t_9_ = 2.77; *p* = 0.02). The analysis of variance showed significant time (*p* = 0.001) and interaction (treatment × time) (*p* = 0.007) effects. Treatment effect showed a tendency toward significance (*p* = 0.09).
[40]	Healthy control vs. ET patients	50 Hz cTBS triplets every 200 ms at 80% AMT for 10 days	M1	10	FTMTRS	Neither the real cTBS with 80% AMT nor the control cTBS with 30% AMT resulted in a significant reduction in the clinical tremor score (*p* > 0.1).
[41]	Single-blinded, randomized, controlled pilot	1200 daily pulses of 1 Hz rTMS at 90% rMT for 10 days	Cerebellum	20	FTMTRS	No significant difference in functional disability at any point in time (*p* > 0.05). There were no statistically significant differences in FTM Part A, Part B, and Part C scores and total scores of patients in the propranolol group on days 5 and 10 compared with before treatment (*p* > 0.05).
[37]	Double-blinded, sham-controlled, crossover, add-on clinical trial	900 daily pulses of 1 Hz rTMS at 90% rMT for 5 days	Cerebellum	23	FTMTRS	No significant improvement in the total scores in rTMS compared to the sham stimulation on day 5 (*p* = 0.132), day 12 (*p* = 0.574), or day 30 (*p* = 0.382).
[33]	Open-label trial	900 daily pulses of 1 Hz rTMS at 90% rTMS for 5 days	Cerebellum	11	FTMTRS	Repeated rTMS over the cerebellum significantly improved total and specific (tremor, drawing, functional disability) scores and reduced tremor amplitude (*p* < 0.006).
[39]	Single-blinded, randomized, sham-controlled pilot study	1200 daily pulses of 1 Hz rTMS at 90% rMT for 5 days	Cerebellum	12	FTMTRS	FTMTRS-A and FTMTRS-B, and total FTMTRS repeated ANOVA indicated a significant effect of ‘time’ (df = 2, F = 14.786, *p* < 0.001; df = 2, F = 18.446, *p* < 0.0001; df = 2, F = 26.623, *p* < 0.001, respectively) but no significant effect of ‘group’ (df = 1, F = 1.976, *p* = 0.176; df = 1, F = 2.175, *p* = 0.157; df = 1, F = 2.367, *p* = 0.140, respectively) nor interaction.
